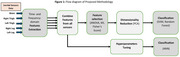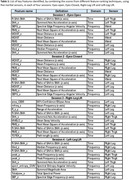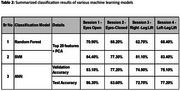# Leveraging Machine Learning Techniques using Inertial Sensor‐based Static Balance Data for Early Detection of Mild Cognitive Impairment

**DOI:** 10.1002/alz70856_106452

**Published:** 2026-01-07

**Authors:** Mobeena Jamshed, Ahsan Shahzad, Kiseon Kim

**Affiliations:** ^1^ National University of Sciences and Technology (NUST), Islamabad, ICT, Pakistan; ^2^ School of Electrical Engineering and Computer Science Gwangju Institute of Science and Technology, Gwangju 61005, Gwangju, Korea, Republic of (South)

## Abstract

**Background:**

Mild Cognitive Impairment (MCI), an early stage of dementia, is often difficult to diagnose due to its subtle and transitional nature. Research has shown that balance impairments can serve as early indicators of cognitive decline, highlighting the potential of wearable sensor technology for detecting MCI at an early stage.

**Method:**

In this study, balance data was collected from 60 participants (30 cognitively normal, 30 with MCI) at the National Research Center for Dementia, South Korea. Shimmer‐3 inertial sensors were placed on the lower back, left and right thigh, left and right legs. Data acquisition involved four conditions: eyes open (EO), eyes closed (EC), right‐leg lift (RL), and left‐leg lift (LL). A total of 76 features were extracted from each sensor, comprising 43 time‐domain and 33 frequency‐domain measures. In the first step, features from all sensors were combined. Extensive feature selection was performed using a combination of different Wrapper methods, namely *ANOVA (Analysis of Variance)*, *Fisher's Score* and *Mutual Information*, followed by dimensionality‐reduction through Principal Component Analysis (PCA). The transformed dataset was subsequently utilized for classification, including *Support Vector Machine (SVM)* and *Random Forest*. Additionally, the extracted features were utilized to perform classification through an Artificial Neural Network (ANN) model. These models were evaluated using Leave‐One‐Subject‐Out (LOSO) cross‐validation technique.

**Result:**

Key time‐domain features, such as *mean‐distance*, *root‐mean‐square‐of‐acceleration*, *range* and *summed‐axis‐acceleration*, alongside frequency‐domain features like *median*, *peak* and *mean* frequency, were identified as significant balance features. In eyes‐open condition, SVM achieved classification accuracy of 84%, whereas 83% validation accuracy and 86% test accuracy were obtained by ANN. Therefore, balance characteristics in eyes‐open condition were proven to be highly distinctive, providing promising opportunities for early dementia detection.

**Conclusion:**

The proposed balance biomarkers derived from inertial sensors demonstrate the potential for early detection of MCI, paving the way for non‐invasive screening solutions to facilitate timely intervention in dementia care.